# Associations between systemic immune-inflammation index and heart failure: A cross-sectional study

**DOI:** 10.1097/MD.0000000000040096

**Published:** 2024-10-18

**Authors:** Zhenkun He, Bizhen Gao, Yuzhou Deng, Juncheng Wu, Xianhui Hu, Zhongxin Qin

**Affiliations:** a Department of Cardiology, Suizhou Hospital, Hubei University of Medicine, Suizhou, Hubei, People’s Republic of China; b Department of Cardiology, The First Affiliated Hospital, Hubei University of Medicine, Shiyan, Hubei, People’s Republic of China.

**Keywords:** cardiovascular and cerebrovascular diseases, heart failure, inflammations, NHANES, systemic immune-inflammation index

## Abstract

The detrimental effects of inflammation on cardiovascular health have received a lot of attention. However, the relationship between heart failure (HF) and the systemic immune-inflammation index (SII) has not been demonstrated. The authors sought to learn more about the relationship between HF and SII in US adults. Adults with complete SII and HF information from the 1999 to 2018 National Health and Nutrition Examination Survey participated in the current cross-sectional study. The calculation for SII involved multiplying the platelet count by the neutrophil count and then dividing it by the lymphocyte count. The relationship between SII and HF was studied using multivariate logistic regression, sensitivity analysis, and smoothed curve fitting. A total of 49,471 participants were enrolled in the study, and 1625 patients (3.28%) were diagnosed with HF. In the model that took all relevant factors into account, we observed that for every 100-unit increase in SII, there was a 2% higher likelihood of developing HF (OR = 1.02; 95% CI: 1.01–1.03, *P* < .0016). Furthermore, we discovered L-shaped associations between SII levels and HF. In subgroups stratified by smoking and diabetes, SII was found to be substantially associated with HF (*P* < .05). Interaction tests revealed that this positive association was not significantly influenced by gender, age, body mass index, smoking status, diabetes, or hypertension (all *P* for interaction > 0.05). In US adults, SII and HF had a positive association. Our study suggests that SII may be a convenient and readily available marker for identifying HF.

## 1. Introduction

Heart failure (HF) is a complex clinical syndrome with symptoms and signs that result from any structural or functional impairment of ventricular filling or ejection of blood.^[1]^ The prevalence of HF has been increasing in recent years, and the prevalence of heart failure increases significantly with age, the incidence of HF is generally estimated to be 1 to 20 cases per 1000 population.^[2]^ HF is a serious and growing public health burden worldwide, but its incidence will increase due to an aging population and improved treatment, leading to further increases in hospitalization rates and a significant burden on healthcare systems.^[3,4]^ Despite advances in treatment and the use of additional interventions, mortality and readmission rates for patients with heart failure have not been significantly reduced.^[5–7]^ A deeper comprehension of the connection between risk factors and heart failure is necessary to prevent the development and progression of heart failure.

The calculation of the systemic immune-inflammation index (SII) involves multiplying the platelet count by the ratio of neutrophil count to lymphocyte count.^[8,9]^ The SII is an innovative measure of inflammation that can be utilized to evaluate the extent of systemic inflammation and the condition of the immune system.^[10,11]^ According to previous research, there is evidence to suggest that SII could potentially serve as an indicator for predicting mortality in patients with cancer.^[12–14]^ Recent research has indicated that SII is a significant factor in diagnosing hypertension, atrial fibrillation, and atherosclerotic conditions.^[15–18]^

SII plays a role in diagnosing and prognostic assessing cardiovascular disease, while inflammation is considered an important factor contributing to HF.^[19]^ As a result, we utilized data from the National Health and Nutrition Examination Survey (NHANES) spanning from 1998 to 2018 in order to conduct a cross-sectional analysis investigating the correlation between SII and HF.

## 2. Methods

### 2.1. Survey description

The authors gathered information from the National Health and Nutrition Examination Assessment (NHANES), a cross-sectional, national, population-based assessment of nutrition and health status in the United States performed by the National Center for Health Statistics (NCHS).^[20,21]^ Because complicated multistage stratified probability sampling was used and it was done on a biannual basis, the samples were representative.

### 2.2. Study population

**Figure 1.**
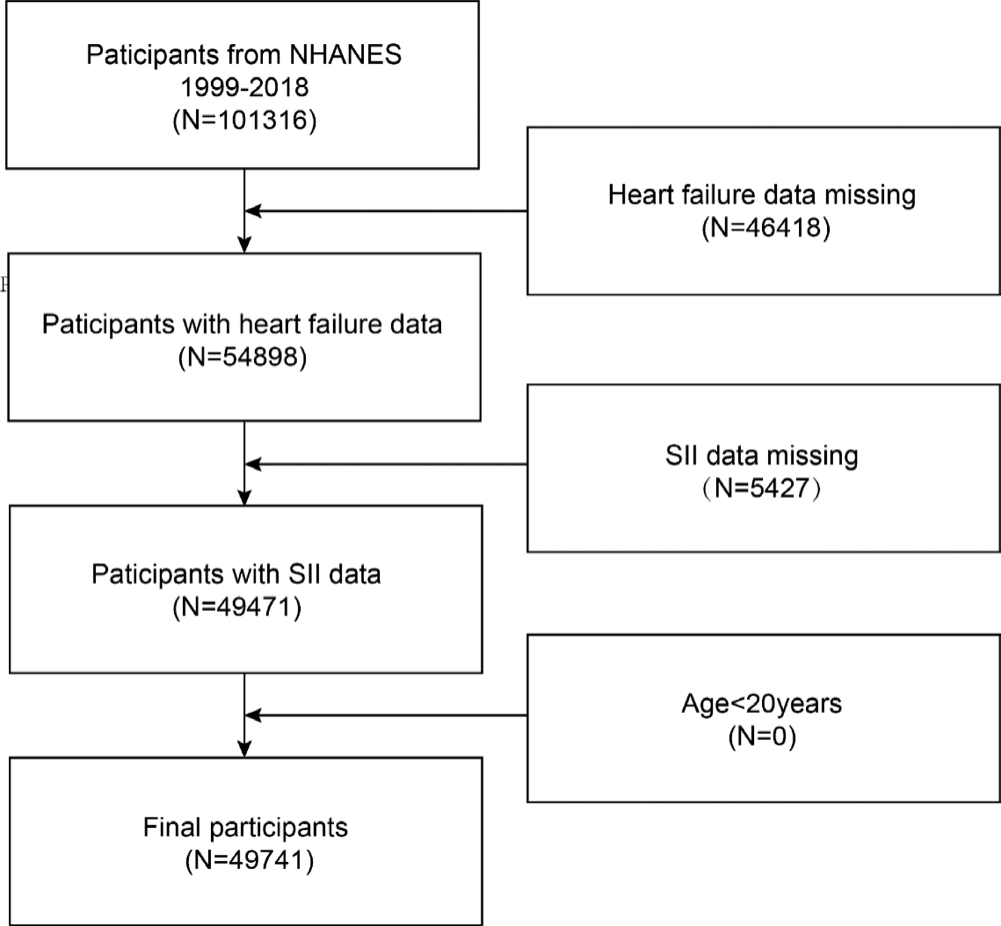
Flowchart of the sample selection from NHANES 1998 to 2018.

This study is based on the 1999 to 2018 NHANES survey cycles. The same variables are used to calculate the SII and assess HF. Exclusion criteria included: age < 20 years; missing HF status; and missing SII data. A final number of 101,316 participants were initially recruited; after excluding participants with missing data on HF status (n = 46,418), SII(n = 5427), 49,741 were eligible (Fig. 1).

### 2.3. Assessment of heart failure

Participants were asked in the health questionnaires “whether a doctor or other health professional has ever told you that you had HF,” and those who answered “yes” were deemed to have HF, similar to prior NHANES-based publications that have been published.^[22]^

### 2.4. Definition of systemic immune-inflammation index

In this research, we focused on the systemic immunity-inflammation index as the dependent variable. The SII level was determined by multiplying the platelet count by the neutrophil/lymphocyte count.^[23,24]^

### 2.5. Covariates

Covariates that may influence the relationship between SII and HF are also included in our study, including gender (male/female), age (year), race (Mexican American/other Hispanic/non-Hispanic White/non-Hispanic Black/other races), family income-to-poverty ratio (PIR), education level (less than high school/high school/more than high school), smoking status (smoking is defined as having smoked more than 100 cigarettes in a lifetime), body mass index (BMI, kg/m^2^), triglyceride (TG, mmol/L), waist circumference (WC, cm), hypertension (yes/no), diabetes (yes/no), coronary heart disease (yes/no). The participants provided self-reported data during the home interview, including information on their gender, age, race, PIR, education level, smoking status, BMI, WC, hypertension, diabetes, and coronary heart disease. In addition, we collected TG data from the laboratory tests.

### 2.6. Statistical analysis

By dividing the participants into SII quartiles, we examined their demographics using chi-squared and t-tests. We also conducted weighted multivariate linear and logistic regression analyses to investigate any linear connections between SII and HF. Model 1 did not adjust for covariates. In model 2, we made adjustments for gender, age, and race. In model 3, age, sex, race, PIR, education, smoking, TG, BMI, WC, hypertension, diabetes, and coronary heart disease were adjusted. The association between SII and HF in individuals with different sex, BMI, education, smoking, hypertension, and diabetes status was examined using subgroup analysis, and interaction tests were used to assess whether the relationships were consistent in the subgroups. Smoothing curve fitting was used to explore the nonlinear association between SII and HF, and a recursive algorithm was used to calculate the inflection point. Then, the 2-piecewise linear regression model was used to analyze the relationship between SII and HF on both sides of the inflection point. We performed all analyses using R (version 4.2) or Empowerstats (version 4.1). The level of statistical significance was set at *P* < .05.

## 3. Results

### 3.1. Baseline characteristics of participants

The study included a total of 49,471 individuals. Of these participants, 48.1% were male and 51.9% were female. On average, the participants were 49.68 ± 18.16 years old. Among the study population, there were 1625 patients diagnosed with HF, which represents 3.28% of the total. Table 1 displays the clinical characteristics of the participants categorized into quartiles based on SII. The table reveals statistically significant differences in age, gender, race, education level, income, body mass index (BMI), WC, smoking status, presence of hypertension, coronary heart disease, diabetes, triglyceride levels, and HF among the different SII quartiles (*P* < .05).

**Table 1:** Baseline characteristics of participants.

Characteristics	Systemic immune-inflammation index				*P*-value
	Q1 (N = 12368)	Q2 (N = 12366)	Q3 (N = 12369)	Q4 (N = 12368)	
Age (yr)	49.82 ± 17.75	49.46 ± 17.70	49.42 ± 18.04	50.02 ± 19.14	.025
Gender (%)					
Male	54.03	50.11	46.82	41.43	<.001
Female	45.97	49.89	53.18	58.57	
Race (%)					
Mexican American	14.77	18.45	19.27	18.13	<.001
Other Hispanic	8.19	8.73	8.53	7.57	
Non-Hispanic White	33.17	44.02	47.83	53.17	
Non-Hispanic Black	32.98	18.80	16.01	13.62	
Other Races	10.88	10.00	8.36	7.52	
Education level (%)					
<High school	27.52	26.94	26.99	26.46	<.001
High school	50.55	50.20	51.82	53.48	
>High school	21.93	22.85	21.19	20.06	
PIR	2.53 ± 1.62	2.59 ± 1.64	2.56 ± 1.63	2.47 ± 1.60	<.001
BMI (kg/m^2^)	28.34 ± 6.28	28.75 ± 6.37	29.23 ± 6.78	29.57 ± 7.62	<.001
WC (cm)	97.00 ± 15.44	98.29 ± 15.42	99.44 ± 16.06	100.39 ± 16.93	<.001
Smoke (%)					
Yes	43.32	44.30	45.87	48.89	<.001
No	56.68	55.70	54.13	51.11	
Hypertension (%)					
Yes	34.14	32.92	34.60	37.06	<.001
No	65.86	67.08	65.40	62.94	
CHD (%)					
Yes	4.00	3.72	4.17	4.60	.005
No	96.00	96.28	95.83	95.40	
Diabetes (%)					
Yes	11.63	11.96	11.58	13.28	<.001
No	88.37	88.04	88.42	86.72	
Triglyceride (mmol/L)	1.42 ± 1.27	1.50 ± 1.17	1.52 ± 1.15	1.57 ± 1.08	<.001
Heart failure (%)					
Yes	3.06	2.59	3.12	4.37	<.001
No	96.94	97.41	96.88	95.63	

Q1 = <341.9, Q2 = 341.9 to 481.8, Q3 = 481.9 to 682.9, Q4 = >683.

BMI = body mass index, CHD = coronary heart disease, PIR = the ratio of income-to poverty, WC = waist circumference.

### 3.2. The association between systemic immune-inflammation index and HF

**Figure 2.**
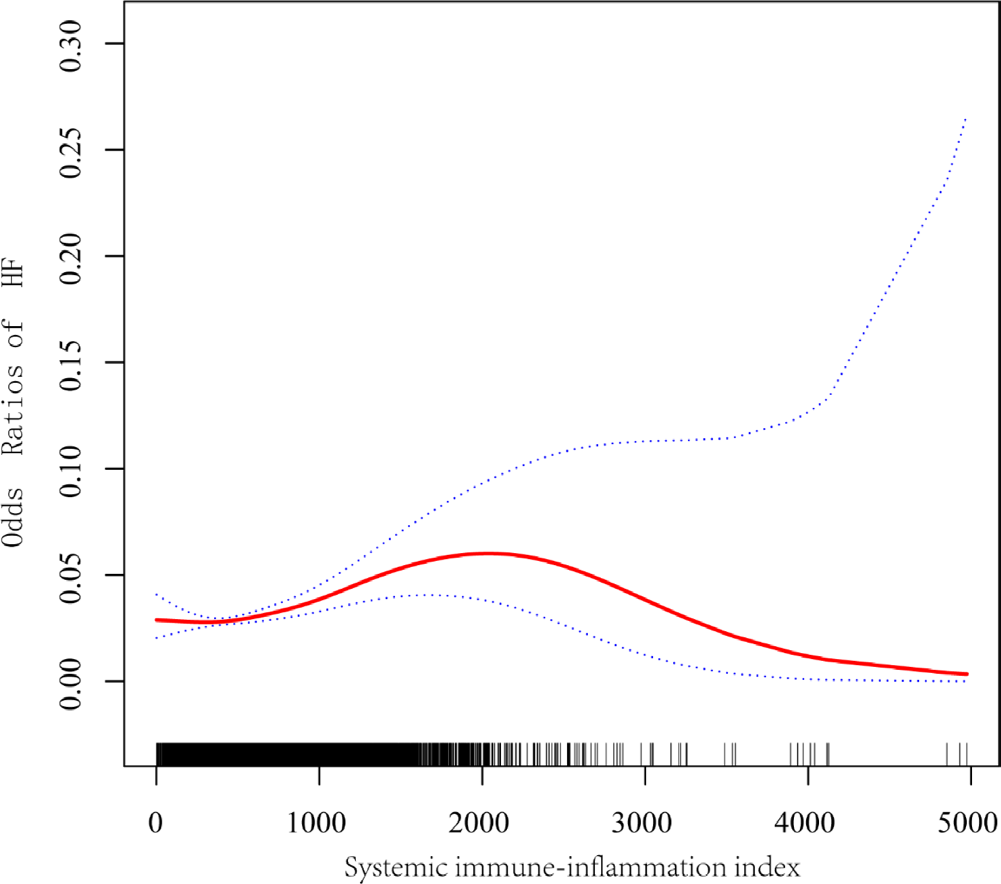
A smooth curve fitting for the relationship between SII and heart failure (HF). Adjust for: age, sex, race, PIR, education, smoking, TG, BMI, WC, hypertension, diabetes, and coronary heart disease. The solid red line represents the smooth curve fit between the variables, while the blue bands indicate the 95% confidence interval from the fit.

Table 2 presents the relationship between SII and HF, and we examine the linear relationship between SII and HF per 100 units because the impact per unit of SII on HF is slight. Our findings indicate that higher SII levels may be linked to an elevated likelihood of developing HF. This connection was statistically significant in both our model 1 (OR = 1.04; 95% CI: 1.04–1.05, *P* < .0001) and model 2 (OR = 1.03; 95% CI: 1.02–1.04, *P* < .0001). In model 3, the positive relationship between SII and HF was maintained (OR = 1.02; 95% CI: 1.01–1.03, *P* < .0016), and the likelihood of developing HF rises by 2% with every 100-unit increase in SII. We took the SII, which was initially a continuous variable, and converted it into quartiles for sensitivity analysis. When comparing participants in the highest SII quartile with those in the lowest quartile, we found that the risk of HF increased by 39% with statistical significance (OR = 1.39; 95% CI: 1.09–1.77, *P* = .0010). Furthermore, the outcomes of the smoothed curve fitting confirm the existence of a relationship between SII with HF as an L-shaped curve (Fig. 2). The impacts of inflection points and 2 segments in the inverted U-shaped relationship were then further examined using threshold effects analysis and 2-segment linear regression models. The results showed that the inflection points of SII for HF were 1144, when the SII level is <1144, the risk of HF increases by 6 percent for each 1-unit increase in the SII level (HR = 1.06; 95% CI: 1.03–1.09), while SII levels >1144 were not statistically associated with HF risk (Table 3).

**Table 2:** Association between systemic immune-inflammation index and HF.

SII/100	Model 1 (OR [95% CI])	Model 2 (OR [95% CI])	Model 3 (OR [95% CI])
Continuous	1.04 (1.04, 1.05); <0.0001	1.03 (1.02, 1.04); <0.0001	1.02 (1.01, 1.03); 0.0016
Categories			
Quartile 1	1 (ref)	1 (ref)	1 (ref)
Quartile 2	0.84 (0.72, 0.98); 0.0263	0.90 (0.77, 1.06); 0.2011	0.92 (0.71, 1.20); 0.5468
Quartile 3	1.02 (0.88, 1.18); 0.7696	1.09 (0.94, 1.26); 0.2792	1.11 (0.86, 1.43); 0.4215
Quartile 4	1.45 (1.27, 1.66); <0.0001	1.42 (1.24, 1.64); <0.0001	1.39 (1.09, 1.77); 0.0080
*P* for trend	<.0001	<.0001	.0010

Model 1: no covariates were adjusted. Model 2: gender, age, and race were adjusted. Model 3: age, sex, race, PIR, education, smoking, TG, BMI, WC, hypertension, diabetes, and coronary heart disease were adjusted.

CI = confidence interval, OR = odd ratio.

**Table 3:** Threshold effect analysis of SII in HF.

	Adjusted HR (95% CI), *P*-value
Heart failure	
Fitting by the standard linear model	1.02 (1.01, 1.03), <.01
Fitting by the 2-piecewise linear model	
Inflection point	1144
SII < 1144	1.06 (1.03, 1.09), <.01
SII > 1144	1.01 (0.99, 1.02), .27
*P* for Log-likelihood ratio	.010

### 3.3. Subgroup analysis

As demonstrated in Table 3, upon conducting additional subgroup analysis, it was observed that the association between SII and HF varied in a non-uniform manner. In subgroups stratified by smoking and diabetes, SII was observed to substantially correlate with HF (*P* < .05). The findings from the interaction tests suggest that factors such as gender, age, BMI, smoking status, diabetes, and hypertension did not have a significant effect on the relationship between SII and HF. Hence, these variables did not significantly influence the positive correlation observed between SII and HF (p for interaction > 0.05) (Table 4).

**Table 4:** Subgroup analysis of the association between SII/100 and HF.

Subgroup	Heart failure [OR (95%CI)]	*P* for interaction
Gender		
Male	1.02 (1.00, 1.03)	.564
Female	1.02 (0.99, 1.05)	
Age		
<60 yr	1.00 (0.95, 1.06)	.593
≥60 yr	1.02 (1.01, 1.03)	
BMI		
<25 kg/m^2^	1.03 (1.00, 1.07)	.460
25 to 29.9 kg/m^2^	1.02 (1.00, 1.03)	
≥30 kg/m^2^	1.01 (0.97, 1.04)	
Smoking		
Yes	1.03 (1.01, 1.05)	.292
No	1.01 (1.00, 1.03)	
Diabetes		
Yes	1.03 (1.01, 1.06)	.251
No	1.01 (1.00, 1.03)	
Hypertension		
Yes	1.02 (1.01, 1.03)	.899
No	1.02 (0.97, 1.07)	

Age, sex, race, PIR, education, smoking, TG, BMI, WC, hypertension, diabetes, and coronary heart disease were adjusted.

## 4. Discussion

Our 49,741-person cross-sectional study revealed that individuals with higher SII had a higher chance of having HF. Furthermore, we discovered L-shaped associations between SII levels and HF. Subgroup studies and interaction tests showed that this association was consistent across populations. According to our data, elevated SII levels are an independent risk factor for HF.

As far as we know, this is the first study that examines the relationship between SII and HF. The immune system and inflammation have a significant impact on the development of atherosclerosis, which is a key contributor to various cardiovascular events.^[25,26]^ In a 30-patient clinical trial, the findings demonstrated a notable correlation between elevations in high-sensitivity C-reactive protein, interleukin-6, and monocytes, and the incidence of acute HF and congestive HF.^[27]^ The findings from a study involving 985 individuals diagnosed with coronary artery disease indicated that elevated levels of CRP (C-reactive protein) at the commencement of the research were linked to a higher probability of the development of HF.^[28]^ In the study conducted by Suleiman M, we discovered a significant correlation between the levels of CRP in individuals who experienced acute myocardial infarction and the subsequent development of HF and long-term mortality, the C-reactive protein to albumin ratio can also be used for prognostic evaluation in HF patients.^[29,30]^ In a research conducted by Getawa S, the primary hematological irregularities noted in individuals with HF were an elevated count of neutrophils and lymphocytes, along with a decreased count of platelets.^[31]^ Durmus E conducted additional research to examine the correlation between the neutrophil-to-lymphocyte ratio (NLR) and platelet-to-lymphocyte ratio (PLR) with HF. Their findings revealed that patients diagnosed with HF exhibited elevated levels of NLR and PLR.^[32,33]^ Some value exists for serum albumin and diagnostic nutritional indices in predicting long-term mortality in HF.^[34–36]^ In addition, a study of 3784 patients with atrial fibrillation showed that biomarkers of inflammation strongly predicted HF hospitalization.^[37]^ Our current study has found a positive relationship between SII and HF, consistent with previous research.

SII was initially suggested as a biomarker to evaluate the outlook of individuals with hepatocellular carcinoma and subsequently employed in research pertaining to tumor detection and prognosis evaluation.^[10,38]^ However, a study conducted by Ye found that the Simplified Index of Inflammation (SII), which is readily available, is highly effective in detecting cardiovascular disease.^[39]^ In a research study that encompassed a cohort of 4606 individuals diagnosed with HF, it was observed that a significant elevation in SII levels could serve as a reliable indicator for predicting high mortalities within 30 and 90 days of hospitalization.^[40]^ In addition, a retrospective study that included 1011 patients showed that SII could potentially serve as a reliable indicator for predicting both long-term mortality and the need for appropriate intracardiac defibrillator therapy in patients diagnosed with HF and reduced ejection fraction.^[41]^ According to a study conducted by Wang, it was shown that SII has emerged as a novel factor that has the ability to forecast the likelihood of mortality in patients who suffer from advanced chronic HF and also have renal dysfunction.^[42]^ The results of our subgroup analysis also indicate that there may be a more noticeable impact of SII in older adults aged 60 years and above who have HF.

The precise mechanism underlying the link between inflammation and HF is not fully understood. Inflammatory cytokines may contribute to the incidence and progression of HF by stimulating the growth of heart muscle cells, activating enzymes that break down the extracellular matrix, causing impaired contractile function, and triggering cell death.^[43–46]^ Neutrophils have a significant impact on the body’s response to increased pressure in the heart and the resulting impaired heart function.^[47]^ Platelets may promote myocardial fibrosis and dysfunction by regulating thrombogenicity, inflammation, endothelial dysfunction, and oxidative stress.^[48]^ On the contrary, lymphocytes possess the ability to regulate the inflammatory response and exert a beneficial effect against atherosclerosis.^[49]^

There are multiple advantages to our study. The foundation of our study is the NHANES data, which ensures that the sample selection is indicative of the entire population and that the sample size is adequately significant. Additionally, we took into account the confounding covariates to guarantee the dependability of our findings. However, our research does have a few constraints. Primarily, due to the nature of our study being cross-sectional, we were unable to definitively establish a cause-and-effect connection between SII and HF. Therefore, future research with larger samples and a study design that looks ahead is needed to better establish a clear causal relationship. Even after accounting for certain variables, we were unable to eliminate the potential influence of other factors that could affect the results.

## 5. Conclusion

Our research has shown a positive correlation between elevated SII and HF. We further discovered inverted L-shaped associations between SII levels and HF. However, it is important to conduct larger-scale prospective studies to validate our findings.

## Author contributions

**Conceptualization:** Zhenkun He, Bizhen Gao, Juncheng Wu.

**Formal analysis:** Bizhen Gao.

**Investigation:** Juncheng Wu.

**Methodology:** Zhenkun He, Bizhen Gao, Zhongxin Qin.

**Project administration:** Zhenkun He, Juncheng Wu.

**Software:** Zhenkun He.

**Supervision:** Yuzhou Deng, Zhongxin Qin.

**Validation:** Bizhen Gao, Yuzhou Deng, Xianhui Hu.

**Visualization:** Xianhui Hu.

**Writing – original draft:** Zhenkun He.

**Writing – review & editing:** Bizhen Gao, Zhongxin Qin.
